# The Role of SHBG as a Marker in Male Patients with Metabolic-Associated Fatty Liver Disease: Insights into Metabolic and Hormonal Status

**DOI:** 10.3390/jcm13247717

**Published:** 2024-12-18

**Authors:** Ljiljana Fodor Duric, Velimir Belčić, Anja Oberiter Korbar, Sanja Ćurković, Bozidar Vujicic, Tonko Gulin, Jelena Muslim, Matko Gulin, Mladen Grgurević, Edina Catic Cuti

**Affiliations:** 1 School of Medicine, University of Catholica Croatica, 10000 Zagreb, Croatia; 2 Department of Nephrology and Arterial Hypertension, Medikol Polyclinic, 10000 Zagreb, Croatia; 3 Medikol Polyclinic, 10000 Zagreb, Croatia; velimir.belcic@medikol.hr (V.B.); jelena.muslim@medikol.hr (J.M.); 4 CAIR-Centar, The House of Statistics, 10000 Zagreb, Croatia; anja.oberiter-korbar@cair-center.hr; 5 Faculty of Kinesiology, University of Zagreb, 10000 Zagreb, Croatia; sanja.curkovic@gmail.com; 6 Faculty of Agriculture, University of Zagreb, 10000 Zagreb, Croatia; 7 School of Medicine, University of Rijeka, 10000 Rijeka, Croatia; vujicic.bozidar@gmail.com; 8 Department of Nephrology, Dialysis and Transplantation, University Hospital Center Rijeka, 10000 Rijeka, Croatia; 9 School of Medicine, University of Zagreb, 10000 Zagreb, Croatia; tonko.gulin@gmail.com; 10 Department of Nephrology and Arterial Hypertension, University Hospital Center “Sestre Milosrdnice”, 10000 Zagreb, Croatia; 11 Department of Radiology, University Hospital Center “Sestre Milosrdnice”, 10000 Zagreb, Croatia; matko.gulin7@gmail.com; 12 Department of Diabetes, Endocrinology and Metabolic Diseases Vuk Vrhovac, Merkur University Hospital, 10000 Zagreb, Croatia; grgurevic.mladen@gmail.com; 13 Croatia Polyclinic, 10000 Zagreb, Croatia; edina3110@gmail.com

**Keywords:** Metabolic-Associated Fatty Liver Disease (MAFLD), sex hormone-binding globulin, insulin resistance, physical activity, cardiovascular health

## Abstract

**Background**: Metabolic-associated fatty liver disease (MAFLD) is a spectrum of liver diseases linked to insulin resistance (IR), type 2 diabetes, and metabolic disorders. IR accelerates fat accumulation in the liver, worsening MAFLD. Regular physical activity and weight loss can improve liver function, reduce fat, and lower cardiovascular risk. This study examines the role of sex hormone-binding globulin (SHBG) in MAFLD, focusing on its potential as a biomarker and its relationship with insulin resistance. **Methods**: The study included 98 male patients (ages 30–55) with MAFLD, identified through systematic examinations, and 74 healthy male controls. All participants underwent abdominal ultrasound and blood tests after fasting, assessing markers such as glucose, liver enzymes (AST, ALT, γGT), lipids (cholesterol, triglycerides), insulin, SHBG, estradiol, and testosterone. SHBG levels were analyzed in relation to body mass index (BMI) and age. **Results**: A significant association was found between low SHBG levels and the presence of fatty liver. Individuals with MAFLD had lower SHBG levels compared to controls. BMI and age were key factors influencing SHBG, with higher BMI linked to lower SHBG in younger men, while SHBG remained stable in older individuals regardless of BMI. **Conclusion**: SHBG may serve as a valuable biomarker for early detection and risk assessment of MAFLD. The complex relationship between SHBG, BMI, and age highlights the importance of considering both hormonal and metabolic factors when assessing fatty liver risk. Our findings support the need for comprehensive metabolic evaluations in clinical practice.

## 1. Introduction

Metabolically Associated Fatty Liver Disease (MAFLD) is increasingly recognized as a global public health concern. Its rising prevalence is closely linked to the growing rates of obesity and sedentary lifestyles [1,2]. The term MAFLD, which emphasizes the metabolic component of the disease, has replaced the older terminology Non-Alcoholic Fatty Liver Disease (NAFLD), shifting focus from the exclusion of alcohol to the inclusion of metabolic health parameters [3]. Insulin resistance (IR) is a key factor that accelerates fat accumulation in the liver, worsening MAFLD and increasing the risk of cardiovascular complications [4,5].

Recent research has explored the potential role of sex hormone-binding globulin (SHBG) in metabolic disorders, given its association with insulin resistance (IR) and liver fat accumulation [6]. Low SHBG levels have been linked to metabolic dysfunction, suggesting a potential role as an indicator of fatty liver risk [7,8].

This study aimed to evaluate the relationship between SHBG and MAFLD in a male cohort and to explore interactions with body mass index (BMI), age, and other metabolic factors.

## 2. Androgen Dysfunction and Metabolic Associated Fatty Liver Disease—The Role of Sex Hormone-Binding Globulin (SHBG)

Obesity and metabolic syndrome contribute to the onset of MAFLD and are often associated with endocrine and hormonal imbalances. The liver plays a crucial role in metabolic processes related to sexual dimorphism [9,10,11]. The prevalence of MAFLD is 2.0–3.5 times higher in men compared to women [12], and epidemiological data indicate that the condition is more severe in men. This suggests a detrimental impact of androgens, while estrogens may exert a protective effect in the development of MAFLD [13,14,15]. However, the exact sex-specific mechanisms driving the development and progression of MAFLD remain unclear. Sex-based differences in the prevalence, progression, outcomes, and comorbidities of fatty liver may reflect the distinct liver phenotypes observed between men and women [12]. This sexual dimorphism in MAFLD is particularly evident in conditions such as polycystic ovary syndrome (PCOS) in women and hypogonadism in men. Recent studies have identified SHBG as a potential marker for MAFLD. Further research into the role of androgens in the development and progression of MAFLD, with a focus on these gender-specific differences, is essential.

## 3. The Role of Sex Hormone-Binding Globulin in the Progression of Metabolic Associated Fatty Liver Disease (MAFLD)

SHBG is a glycoprotein produced by the liver [16]. Its primary function is binding and transporting circulating testosterone and estradiol, regulating their bioavailability and sequestering circulating androgens and estrogens [17,18]. SHBG has a high affinity for testosterone and a lower affinity for estradiol [18]. The free testosterone concentration in plasma is heavily influenced by SHBG levels, as only 1–2% of testosterone in plasma is free and active; 65% is bound to SHBG, with the remainder bound to albumin. Additionally, SHBG plays a role in signal transduction.

An experimental study demonstrated that thyroid and estrogen hormones enhance SHBG synthesis by upregulating the expression of hepatocyte nuclear factor-4α (HNF-4α), a key regulator of SHBG promoter activity in the liver [19,20]. In contrast, PPAR-γ competes with HNF-4α for binding sites on the SHBG promoter, inhibiting SHBG expression [21]. SHBG levels are inversely correlated with hepatic triglycerides and acetyl-CoA carboxylase (ACC) activity [22]. SHBG may inhibit the phosphatidylinositol 3-kinase (PI3K)/protein kinase B (AKT) pathway, which plays a role in the development of both local and systemic insulin resistance (IR) [23]. Increased hepatic lipogenesis or IR reduces HNF-4α expression, decreasing hepatic SHBG synthesis. Additionally, inflammatory conditions influence SHBG levels. In chronic inflammatory diseases, elevated levels of cytokines such as IL-1 and TNF-α impact SHBG production. The effect of IL-1 is mediated through NF-κB, which suppresses HNF-4α transcription, leading to reduced SHBG synthesis [24]. Low testosterone is linked to an unfavorable fat distribution and adipocyte insulin resistance, which reduces the ability to suppress lipolysis, leading to ectopic fat accumulation and “lipotoxicity” [25]. Inflammatory cytokines from adipose tissue, such as TNF-α, IL-6, and C-reactive protein, can disrupt hepatic insulin signaling and promote fat buildup in the liver. This results in the inhibition of HNF-4α mRNA through NF-κB activation or via the Methyl ethyl ketone-1/2 (MET-1/2) and c-Jun N-terminal kinase (JNK) mitogen-activated protein kinase (MAPK) pathways [26,27]. On the other hand, adiponectin stimulates SHBG production by activating AMPK, which enhances fatty acid oxidation and boosts HNF-4α levels [28]. As a result, reduced circulating SHBG is linked to a greater risk of MAFLD in men with hypogonadism.

SHBG has the potential to be a biomarker for MAFLD [29]. Reduced levels of testosterone and SHBG are both associated with metabolic syndrome and fatty liver. According to a recent meta-analysis, low total testosterone levels showed a positive association with MAFLD in men but an inverse relationship in women [29]. Meanwhile, low SHBG levels were consistently linked to a heightened risk of MAFLD development in both genders. SHBG also possesses anti-inflammatory and lipolytic effects on adipocytes and macrophages, which may account for its link to reduced incidence rates of metabolic syndrome and its associated complications [30]. In a study with biopsy-confirmed MAFLD, lower levels of SHBG were found to be inversely related to the severity of steatosis. The potential of enhancing SHBG expression as a therapeutic target for MAFLD is a hopeful prospect that could pave the way for novel treatment options [31].

Male hypogonadism is a clinical syndrome characterized by reduced or absent gonadal function, leading to insufficient testosterone secretion [32]. Obesity is one of the most significant risk factors for secondary hypogonadism in men [33]. Male obesity secondary hypogonadism (MOSH) negatively affects fertility, sexual function, bone mineral density, and fat metabolism, resulting in decreased muscle mass and altered body composition [33]. Although the exact prevalence of MOSH is uncertain, some studies have reported rates as high as 45.0–57.5% [33,34,35]. In a study involving 159 men randomly selected from the NASH Clinical Research Network cohort, 26% of men with MAFLD were found to have low free testosterone [36]. Men with low free testosterone were more likely to exhibit NASH and advanced fibrosis compared to those with simple steatosis (88% vs. 67% and 27% vs. 14%, respectively) [36].

The mechanism that elevates the risk of MAFLD in individuals with hypogonadism has not been well elucidated. Testosterone is crucial for insulin sensitivity, body composition, and lipid metabolism [36]. Low testosterone levels and insulin resistance (IR) [37] have a bidirectional relationship. In preclinical studies, low testosterone levels may cause hepatic fat accumulation through increased DNL via upregulation of hepatic SREBP-1 [38,39]. The upregulation of SREBP-2 and ACC-1 is apparently due to reduced AMP-activated protein kinase (AMPK) activity [38,39]. Testosterone may enhance the expression of hepatic scavenger receptor class B type 1 (SR-B1), which plays a role in the selective uptake of cholesterol esters from circulating high-density lipoproteins (HDL), aiding reverse cholesterol transport. Testosterone downregulates MTTP expression, reducing apolipoprotein B-mediated VLDL secretion and lowering cholesterol 7α-hydroxylase levels. This results in hepatic steatosis, driven by increased cholesterol accumulation and impaired removal [40,41]. Testosterone also notably enhances the mRNA expression of insulin receptors, leading to increased insulin binding and higher glucose oxidation [42]. Additionally, testosterone treatment improves serine phosphorylation of insulin receptor substrate 1 (IRS-1), inhibiting insulin signaling by reducing tyrosine phosphorylation [43,44]. Testosterone deprivation reduces glucose transporter type 4 (GLUT4) expression in liver tissue, leading to hyperglycemia, low insulin levels, and decreased glucose uptake in adipose and skeletal muscle tissue [45]. Additionally, low testosterone and sex hormone-binding globulin (SHBG) levels in men are independent predictors of metabolic syndrome [46]. Low testosterone is associated with visceral fat distribution and shows sexual dimorphism, as fat distribution depends on testosterone in men and estradiol (E2) in women. Testosterone levels are inversely related to visceral fat accumulation. Both testosterone and E2 influence visceral fat expansion by activating estrogen receptors (ERα and ERβ) and androgen receptors (ARs). ER activation occurs through E2 derived from testosterone aromatization; therefore, testosterone deficiency, which leads to reduced estradiol levels, is a critical factor in visceral fat deposition and insulin resistance (IR) in men [47,48,49].

Efficient activation of androgen receptors (AR) reduces body fat and enhances insulin sensitivity [49,50]. Consequently, testosterone plays an anti-obesity role by inhibiting visceral fat accumulation and preventing insulin and leptin resistance, contributing to liver and adipose tissue lipogenesis. These effects are mediated by activating the AR pathway [51,52]. Sex hormone-binding globulin (SHBG) is linked to low testosterone levels in men with adult-onset hypogonadism [53]. SHBG influences testicular negative feedback either directly or by regulating the cellular entry of testosterone or estradiol in the hypothalamus and pituitary, thereby controlling gonadotropin synthesis and secretion [54]. Low total testosterone and SHBG levels are strongly associated with an increased risk of metabolic syndrome independent of insulin resistance (IR) [55]. Thus, hypogonadism is a recognized risk factor for the development of MAFLD. Testosterone replacement therapy in hypogonadal men with metabolic syndrome has beneficial effects on hepatic steatosis, insulin sensitivity, and glucose regulation [56,57]. However, there is currently insufficient evidence to support the use of testosterone therapy, specifically in hypogonadal patients with MAFLD. Furthermore, the biochemical mechanisms underlying the potential therapeutic benefits of testosterone in MAFLD remain to be elucidated, and further study is needed to understand the liver-specific role of testosterone. Studies with large cohorts are necessary to determine whether men with low androgen levels on long-term testosterone therapy are protected against prostate cancer and have a reduced risk of cardiovascular disease over time.

## 4. Correlation Between Insulin Resistance and Sex Hormone-Binding Globulin (SHBG)

Multiple factors contribute to the development of insulin resistance, including obesity, impaired glucose tolerance, excessive alcohol consumption, smoking, high cholesterol levels, elevated triglycerides, low HDL cholesterol, hyperuricemia, and hypertension [58,59,60]. More than 80% of obese individuals experience insulin resistance at some stage in their lives [61,62,63]. When a person becomes obese, adipocytes increase, leading to more extensive and dysfunctional adipose tissues; this process involves the recruitment of macrophages, which subsequently polarise to a pro-inflammatory state [64,65]. Enlarged adipose tissues secrete excess free fatty acids (FFAs), reactive oxygen species (ROS), and pro-inflammatory cytokines. Excess systemic FFAs and dietary lipids can infiltrate non-adipose organs such as the liver, muscle, and pancreas, leading to ectopic fat deposition and lipotoxicity. The accumulation of toxic lipids disrupts cellular organelles, including mitochondria, endoplasmic reticulum, and lysosomes. This dysregulation of organelles triggers the release of excess ROS and pro-inflammatory signals, contributing to systemic inflammation [66,67]. Chronic low-grade systemic inflammation impairs insulin action within the insulin signaling pathway, disrupts glucose homeostasis, and leads to systemic dysregulation. In general, prolonged obesity and overnutrition contribute to the development of insulin resistance and chronic low-grade systemic inflammation through mechanisms such as lipotoxicity, setting the stage for various clinical conditions. The liver is susceptible and experiences insulin impairment more rapidly than other organs; consequently, hepatic insulin resistance is the initial event that paves the way for peripheral tissue insulin resistance [67,68].

## 5. Physical Activity in Individuals with MAFLD

Regular physical activity, such as aerobic exercises and strength training, has been shown to improve insulin sensitivity and reduce fat accumulation in the liver. According to Zalewski P et al. [68], aerobic exercises decrease intrahepatic lipid content (IHL) in individuals with MAFLD, leading to improved liver function and a reduced risk of disease progression. In addition to aerobic exercises, Research by Dirac et al. [69] has also shown that moderate weight loss of 5–10% reduces liver fat and improves liver function in patients with MAFLD [69,70]. One of the critical interventions in treating MAFLD is physical activity, with aerobic exercise programs of at least 150 min per week being particularly recommended, about 20 min per day [71]. Through several biological mechanisms, physical activity reduces liver fat and inflammatory processes in MAFLD [72,73,74]. Physical activity also plays an essential role in restoring hormonal balance. Hypogonadism and imbalances in sex hormones, such as elevated estradiol and reduced testosterone, are common in men with MAFLD [75]. According to research by Duncan et al. [76], aerobic exercise and strength training increase testosterone levels, reduce estradiol levels, and improve insulin sensitivity, thereby reducing metabolic abnormalities. A combination of aerobic and strength training leads to a reduction in visceral fat, further improving hormonal balance [77,78,79]. According to the guidelines of the American College of Sports Medicine [80], engaging in at least 150 min of moderate aerobic activity per week is recommended to improve liver health. This includes activities such as walking, cycling, swimming, badminton, recreational tennis, group fitness programs, social dances, hiking, various outdoor exercises, and gardening, or 75 min of intense activity like running, more intense swimming or cycling, individual and group fitness programs, resistance exercises, high-intensity interval training (HIIT) with short, intense intervals of exercise such as burpees, jumps, and exercises with varying weights. It is also recommended to include strength training at least twice a week, focusing on large muscle groups, which improves insulin sensitivity and reduces fat accumulation in the liver [80,81]. Combining aerobic exercise and strength training improves mitochondrial function and lipid metabolism [82]. These findings highlight the critical role of physical activity in preventing and treating MAFLD, emphasising the need for an active lifestyle to maintain liver health.

## 6. Materials and Methods

This study included 98 male patients diagnosed with metabolic-associated fatty liver disease (MAFLD), some of whom were identified during routine systematic examinations. The participants were men aged between 30 and 55 years, with a mean age of 43. The inclusion criteria were male gender, age between 30 and 55 years, increased body mass index (BMI), and ultrasound findings indicative of fatty liver. Male participants were selected to enable a focused investigation into the role of testosterone and SHBG in metabolic health, a relatively underexplored area compared to studies in women. All participants in the study consume alcohol in moderate amounts and are not physically active. These factors were considered anamnestically, and since they were uniform across all participants, they were not specifically listed in the tables. Although insulin resistance is common in the type 2 diabetes population, our focus was on participants without diabetes.

The control group consisted of 74 men without fatty liver, as determined by ultrasound performed using the Siemens Acuson Sequoia ultrasound machine. Ultrasound was employed as an initial diagnostic tool to detect the presence of fatty liver based on qualitative changes in liver echogenicity. Specific grading criteria for the severity of steatosis were not used, as ultrasound lacks standardization for precise classification. For a more detailed classification of fatty liver, future studies could incorporate techniques such as elastography or FibroScan.

Patients with MAFLD underwent abdominal ultrasound with a complex abdomen protocol due to increased waist circumference, while the control group underwent standard abdominal ultrasound. Exclusion criteria included female gender, ongoing treatment for malignant diseases, and a diagnosis of diabetes. We assessed insulin resistance and the HOMA index in all participants to better understand metabolic status while excluding patients with type 2 diabetes, who may also have other metabolic disorders such as fatty liver disease.

Estradiol and testosterone levels were assessed at a single point in time to evaluate their association with metabolic parameters. The variability and stability of these hormones over time were not addressed in this study. Future research could explore longitudinal changes in hormone levels to better understand their fluctuating nature and their potential impact on MAFLD.

Although a mixed-gender cohort was initially considered, the decision to focus on male participants was made to explore the specific relationship between SHBG and testosterone in the context of MAFLD. While hormonal imbalances and MAFLD are well-studied in women, there is limited research on the effects of testosterone and SHBG in male populations, particularly without the influence of hormone replacement therapy. The impact of potential confounding factors, such as medication use, was considered during the study design. The majority of participants in the healthy group were not on any medications, and only a very small number in the MAFLD group were receiving treatment. Additionally, all participants had normal glucose values, reducing the likelihood of confounding by metabolic disturbances such as diabetes.

The study enrollment occurred between January 2024 and October 2024. The study was conducted in accordance with the Declaration of Helsinki and approved by the Institutional Ethical Board of Medikol Polyclinic (approval date: 7 August 2024). Informed consent was obtained from all participants prior to inclusion.

### Assessment of Clinical and Biochemical Parameters

A comprehensive medical history was collected from all patients, documenting alcohol consumption, smoking habits, and medication history. Anthropometric measurements, including weight, height, waist and hip circumference, and blood pressure, were recorded. Body mass index (BMI) was calculated using the formula weight (kg) divided by height squared (m^2^).

Blood samples were collected after an overnight fast. Biochemical parameters, including fasting serum glucose, AST, ALT, γGT, creatinine, C-reactive protein (CRP), total cholesterol, triglycerides, HDL cholesterol, and LDL cholesterol, were analyzed using the Cobas Pure-c303 (Roche, Mannheim, Germany) biochemistry analyzer. Additionally, serum levels of insulin, SHBG, estradiol, and total testosterone were measured using electrochemiluminescence immunoassays (ECLIA) on the Cobas Pure-e402 (Roche, Mannheim, Germany) immunochemistry analyzer. Serum concentrations of free testosterone were calculated.

These assessments provide valuable insights into the clinical and biochemical characteristics of patients with MAFLD and underscore the importance of tailored evaluation approaches for this population.

## 7. Statistical Analysis

To investigate the hypothesis that low levels of SHBG (Sex Hormone Binding Globulin) are present in obese male individuals with MAFLD (Metabolic Dysfunction-Associated Fatty Liver Disease) who do not have diabetes, the following methodology was employed: The statistical analysis is structured into three parts. First, descriptive statistics (mean, standard deviation, median, minimum, and maximum value) were used to summarise the key characteristics of the study sample, both overall and by group, based on the presence of fatty liver. The data for the study were collected from two distinct groups based on MAFLD (diagnosed by ultrasound): (a) individuals diagnosed with MAFLD (‘fatty liver’) and (b) those who did not have the condition (‘healthy’). Second, bivariate analysis was used to examine pairs of variables, including correlations, univariate regression, and *t*-tests [83]. Correlation analysis assessed the strength and direction of the linear relationships among continuous variables. In contrast, univariate linear regression was utilized to evaluate how various independent variables influence SHBG levels. Additionally, a *t*-test [83] was conducted to compare the mean SHBG levels among individuals diagnosed with ‘fatty liver’ and those without the condition. Finally, a complete and stepwise general linear model (GLM)was employed to evaluate the relationship between SHBG and a range of potential predictors, including metabolic, liver, hormonal, and demographic factors.

Additionally, stepwise logistic regression [83,84] was used to test the hypothesis that SHBG, while controlling for other factors, can significantly predict the presence of MAFLD.

Variables included in univariate and multivariate models were logarithmically transformed when their distributions deviated from normality. Additionally, variables exhibiting high variance inflation factors (VIF) were excluded from the models due to multicollinearity [85].

In the final part of the article, the role of BMI and blood pressure in MAFLD was investigated using univariate and multiple logistic regression models, pairwise comparisons [85], and table analysis. This additional section aimed to elucidate the relationships between these key health indicators and the prevalence of fatty liver disease, thereby contributing to a better understanding of risk factors associated with MAFLD.

All analyses were performed using SAS^®^ 9.4 (SAS Institute Inc., Cary, NC, USA) [86].

## 8. Results

### 8.1. Descriptive Statistics

The study involved 172 male participants. Table 1 summarises all demographic, hormonal, metabolic, and liver function parameters (variables) collected across the sample.

In terms of hormonal levels, SHBG (Sex Hormone-Binding Globulin) had a mean level of 33.23 (14.19) nmol/L, with a median of 31.15 nmol/L and a range of 8.70 to 95.70 nmol/L. Estradiol levels averaged 124.19 (33.58) pmol/L, with a median of 119.00 pmol/L and a range of 52.00 to 226.00 pmol/L. Free Testosterone levels had a mean of 348.57 (100.31) pmol/L, with a median of 331.20 pmol/L and a range of 117.20 to 729.50 pmol/L. Testosterone levels averaged 16.78 (6.06) nmol/L, with a median of 16.07 nmol/L and a range of 5.25 to 38.55 nmol/L.

Regarding metabolic parameters, BMI (Body Mass Index) averaged 27.49 (4.62) kg/m^2^, with a median of 26.45 kg/m^2^ and a range of 18.90 to 47.32 kg/m^2^, suggesting a predominantly overweight population. A waist Circumference of 93.70 (16.44) cm, with a median of 94.00 cm and a range from 60.00 to 117.20 cm, indicates abdominal obesity.

The median age of participants was 43 years, with a range spanning from 29 to 61 years.

Table 2 summarises descriptive statistics comparing individuals with and without metabolic-associated fatty liver disease (MAFLD). The MAFLD group (N = 98) exhibited higher liver enzyme levels, more excellent insulin resistance, more pronounced obesity (both general and central), and lower SHBG levels compared to the healthy group (N = 74). These results support the strong association between obesity, metabolic dysregulation, and the development of fatty liver disease.

### 8.2. Correlation Analysis

Table 3 presents the results of the correlation analysis for variable pairs with moderate to high correlation coefficients (absolute r > 0.4). The study revealed several significant positive correlations, highlighting solid associations between certain variables. These findings suggest meaningful relationships that warrant further exploration and may indicate underlying patterns or trends in the data. Notably, a robust correlation exists between insulin levels and HOMA2 (0.99754). A strong correlation exists between cholesterol and LDL (0.93627), showing that elevated total cholesterol corresponds with higher LDL cholesterol levels. As expected, there is also a strong relationship between weight and waist circumference (0.92106), as is between weight and body mass index (BMI) (0.89033). A strong correlation was also detected between AST and ALT (0.81024).

Moderate positive correlations were observed between BMI and HOMA2 (0.47077), indicating that higher BMI is linked to more excellent insulin resistance. Similarly, a moderate correlation exists between waist circumference and insulin (0.40570), implying that larger waist circumference is associated with higher insulin levels. Lastly, a moderate correlation between weight and insulin (0.42215) suggests that increased weight correlates with higher insulin levels.

Additionally, these findings highlight how hormonal levels, particularly testosterone and SHBG, are interconnected. Testosterone and SHBG showed a strong correlation (0.74944), suggesting that as testosterone levels increase, SHBG levels also rise.

### 8.3. Univariate Regression

The results from the univariate linear regressions, with SHBG as the dependent and each predictor as an independent variable, are presented in Table 4. SHBG was negatively associated with BMI (*p* = 0.0042), weight (*p* = 0.0025), waist circumference (*p* = 0.0005), ALT (*p* = 0.0052), log-transformed HOMA2 (*p* = 0.0010), log-transformed insulin (*p* = 0.0008), log-transformed triglycerides (*p* < 0.0001), and log-transformed GGT (*p* = 0.0060). A negative significant relationship implies that as the predictor variable increased, SHBG decreased. For example, as is indicated in Table 4 and above, BMI had a negative significant association with SHBG, meaning that individuals with higher BMI levels tend to have lower SHBG concentrations. Similarly, for variables like waist circumference, weight, or log-transformed HOMA2, increases were significantly linked with a decrease in SHBG levels. In the context of SHBG, a negative significant relationship with metabolic markers like BMI, weight, or insulin resistance (HOMA2) could indicate that worsening metabolic health (increased adiposity, insulin resistance) is associated with lower levels of SHBG.

Alternatively, positive associations were observed with estradiol (*p* = 0.0011), free testosterone (*p* = 0.0045), total testosterone (*p* < 0.0001), and HDL cholesterol (*p* = 0.0009). These findings suggest that higher SHBG levels may be linked to improved hormonal balance (higher estradiol and testosterone) and better lipid health (higher HDL cholesterol).

### 8.4. Test

**Figure 1:**
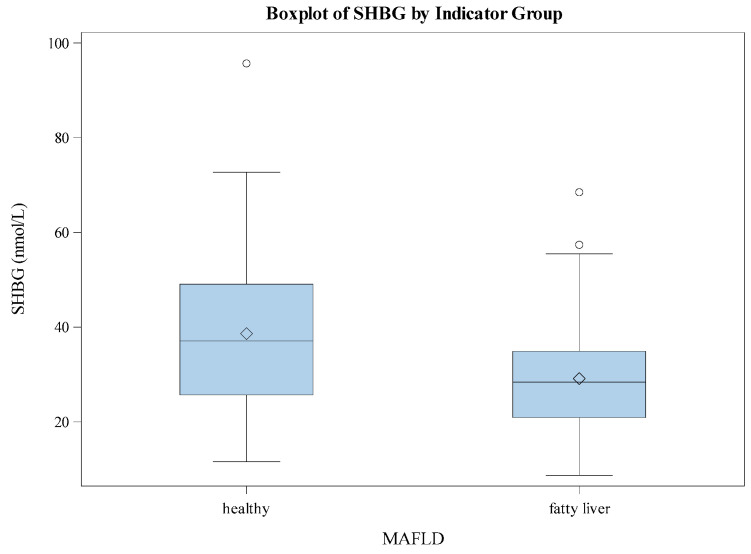
Boxplot of SHBG by MAFLD.

The *t*-test results (Table 5 and Table 6) indicate a statistically significant difference in SHBG levels between the healthy and the group of participants diagnosed with fatty liver. The mean SHBG level was significantly higher in the healthy group compared to the fatty liver group (*p* < 0.0001). The Folded F test for equality of variances suggested that the variances between the two groups were significantly different, with the fatty liver group having less variability in SHBG levels (Table 7). These results emphasize the relationship between SHBG levels and liver health, indicating that individuals with fatty liver have significantly lower SHBG levels than healthy individuals (Figure 1).

### 8.5. General Linear Model

To investigate the potential impact of multiple variables (“predictors”) on SHBG levels, a general linear model (GLM) was applied. The first approach utilized a complete model without employing a variable selection process. This model includes all potential predictors to assess their contribution to the outcome fully; some of the predictors (AST, CRP, GGT, Glucose, Triglycerides, HOMA2) were, due to skewed distributions, logarithmically transformed to “normalize” the data and meet the conditions for statistical testing.

The results of the general linear model, including parameter estimates, standard errors, *t*-values, and *p*-values for all predictors included in the model, are presented in Table 8. These results provide insight into the effect of individual predictors on SHBG levels and their statistical significance after adjusting for the presence of other predictors in the model. In other words, because of the solid and moderate correlations among some of the predictors (shown in Table 3), the results of GLM with 15 predictors (Table 5) differ from those obtained from 15 univariate regression analyses (Table 4).

Fatty liver was found to affect SHBG levels (*p* = 0.0009) significantly negatively. The interaction between BMI and age had a significant positive effect on SHBG (*p* = 0.0191), indicating that the influence of BMI on SHBG may vary with age.

Furthermore, cholesterol levels significantly positively impacted SHBG (*p* = 0.0078), while log-transformed triglycerides were negatively associated with SHBG (*p* = 0.0168). Estradiol levels also significantly positively affected SHBG (*p* = 0.0178). Other predictors, such as prostate volume, HOMA2, HDL, creatinine, log-transformed AST, CRP, GGT, and glucose, did not show significant effects on SHBG levels (*p* > 0.05).

**Figure 2:**
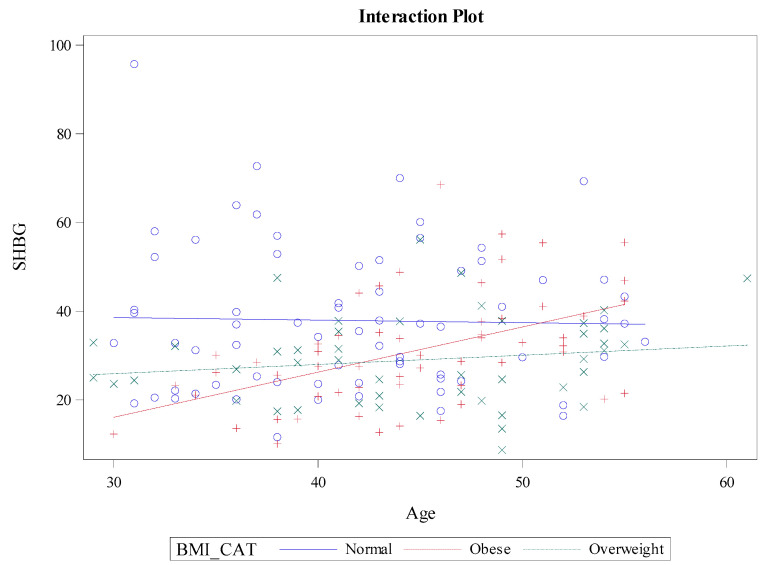
Interaction Between BMI and Age on SHBG Levels.

The interaction between BMI and age, previously detected, is visually represented in Figure 2, with an interaction plot. The plot illustrates that, in older participants, SHBG levels remained relatively constant, regardless of BMI. However, in younger individuals, SHBG levels decreased significantly when the participants were obese, were moderately higher in those who were overweight, and reached the highest levels in individuals with normal BMI. Additionally, for patients with normal BMI, SHBG levels did not vary significantly with age but were relatively constant. For clarity and illustrative purposes, BMI was categorized using WHO standards.

Additionally, in an attempt to identify the most predictive model, a stepwise selection method was applied. This approach sequentially adds or removes variables based on their statistical significance (*p*-values). The Schwarz Bayesian Information Criterion (SBC) was chosen as the stopping criterion. By minimizing SBC, the selection process aims to identify the model that best explains the data with the fewest predictors, ensuring that the final model retains only those variables that contributed meaningfully to the model’s performance [87].

The model identified several significant predictors of SHBG (Table 9 and Table 10), including fatty liver, estradiol, HDL, and free testosterone. All of the predictors, except the presence of fatty liver, demonstrated a positive relationship with SHBG, indicating that higher values were associated with increased SHBG levels. In contrast, the presence of fatty liver again had a significant negative impact on SHBG levels. In this model, the estimate for fatty liver was −12.80 (*p* < 0.0001), indicating that individuals with fatty liver have SHBG levels approximately 12.8 units lower than those without the condition. The significant interaction between BMI and age suggests that their combined effect on SHBG levels was again evident, highlighting the importance of considering these variables together when assessing their influence on SHBG. Overall, the model explains approximately 24.19% of the variance in SHBG levels, reflecting a moderate level of explanatory power, indicating that other factors not included in the model may also play a role.

### 8.6. Logistic Regression

**Figure 3:**
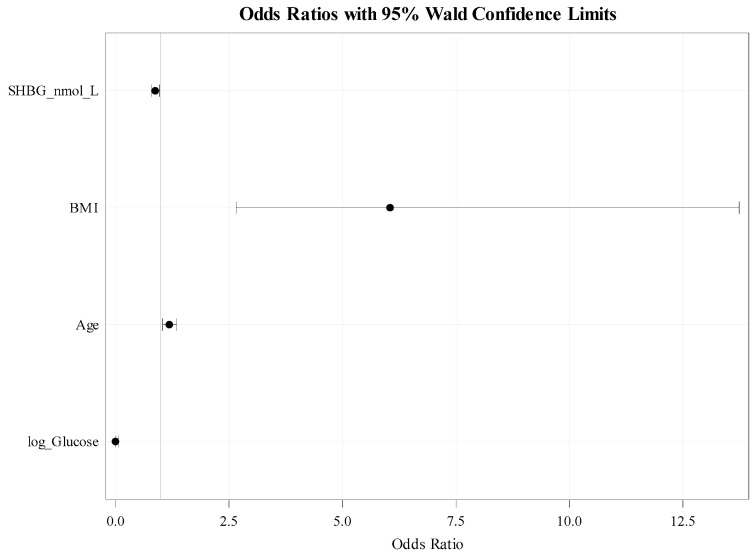
Plot of Odds Ratios and 95% Wald Confide.

The stepwise logistic regression analysis indicates several factors significantly impacted the likelihood of developing fatty liver (Table 11 and Table 12, Figure 3). An increase in one unit of SHBG was associated with a 12.5% reduction in the odds of fatty liver (OR: 0.875, 95% CI: 0.786–0.974). Thus, a decrease in SHBG by five units significantly reduced the odds of developing fatty liver disease. Specifically, the odds decrease to approximately 51.3% of the original odds. This indicates that for every 5-unit decrease in SHBG, the likelihood of developing fatty liver decreased by about 48.7%. BMI was found to be a vital risk factor, with each unit increase in BMI increasing the chances of fatty liver more than sixfold (OR: 6.048, 95% CI: 2.663–13.738), meaning that individuals with higher BMI had a dramatically higher risk of fatty liver. Age also raised the risk, with each additional year of age increasing the odds by 18.6% (OR: 1.186, 95% CI: 1043–1349). Additionally, log-transformed glucose showed a significant protective effect, with a very low odds ratio of <0.001 (95% CI: <0.001–0.065), indicating a substantial reduction in the chances of fatty liver at higher glucose values.

The findings related to the log-transformed glucose levels in the logistic regression model should be interpreted with caution. The study sample consisted exclusively of non-diabetic individuals, and this selection may limit the generalizability of the results to populations with metabolic disorders, such as diabetes.

### 8.7. The Role of BMI and Blood Pressure in Fatty Liver Disease Risk

Logistic regression analysis was employed to investigate the hypothesis that blood pressure is associated with fatty liver disease (MAFLD), as summarized in Table 13.

Initially, univariate logistic regression was conducted to evaluate the unadjusted relationship between each predictor and the likelihood of developing MAFLD.

The analysis (Table 13, Univariate) revealed that systolic blood pressure has a significant association with MAFLD, evidenced by a Wald Chi-Square value of 14.0008 and a *p*-value of 0.0002, indicating that elevated systolic pressure may increase the risk of fatty liver disease. Similarly, diastolic blood pressure demonstrated a strong correlation with MAFLD, with a Wald Chi-Square of 18.6441 and a *p*-value of less than 0.0001, underscoring the role of elevated blood pressure in contributing to disease risk.

Notably, Body Mass Index (BMI) emerged as a strong significant predictor of MAFLD, exhibiting a Wald Chi-Square value of 30.1470 and a *p*-value of less than 0.0001. This indicates a clear and substantial association between higher BMI and an increased likelihood of developing fatty liver disease, reinforcing the importance of managing BMI as a critical factor in mitigating the risk of MAFLD.

However, since these results are derived from univariate regressions with only one predictor at the time in the model, they do not account for potential correlations and interactions among factors, which can limit the understanding of their combined effects on disease risk.

While systolic blood pressure alone can indicate some risk, its significance diminishes (Wald chi-square = 0.3291, *p*-value = 0.5662) when BMI, a more direct measure of metabolic risk is included in the multiple models (Table 13, Multiple (systolic, bmi)). Likewise, when diastolic blood pressure was used as a predictor instead of systolic (Table 13, Multiple (diastolic, bmi)), the results demonstrate that the diastolic blood pressure parameter does not show a statistically significant adjusted relationship with fatty liver, indicated by a high *p*-value (0.4858) and a Wald Chi-Square of only 0.4858. This suggests that diastolic blood pressure may not be a meaningful predictor of fatty liver when controlling for BMI. BMI remains a significant predictor of fatty liver (*p*-value < 0.0001), indicating that as BMI increases, the likelihood of fatty liver also increases.

Finally, in the multiple logistic regression analysis including BMI, diastolic and systolic blood pressure as predictors for fatty liver disease (MAFLD), the results reveal that while the overall model is statistically significant due to the strong contribution of BMI, neither of the two blood pressure measures (diastolic or systolic) provide any indication of additional (adjusted for BMI) predictive power regarding fatty liver disease. This again highlights the dominance of BMI as a primary risk factor for MAFLD, with blood pressure potentially having a secondary role that does not significantly impact the risk in the presence of BMI.

Some patients with high blood pressure were receiving treatment for their condition, prompting an investigation into the potential influence of the therapy. However, due to the absence of significant interaction between blood pressure and therapy, as well as preliminary analyses that failed to demonstrate any meaningful associations, it was determined that therapy did not affect the relationship between blood pressure and the presence of fatty liver in this sample. Consequently, this variable was excluded from the final analysis [88].

To further explore potential trends and interactions within the data, individuals were classified based on specific criteria. Those with a body mass index (BMI) of 25 or above were categorized as overweight, while those with a BMI below 25 were classified as having normal BMI. Similarly, patients with systolic blood pressure readings of 140 mmHg or higher were designated as having high blood pressure (BP), whereas individuals with lower systolic blood pressure were classified as having normal blood pressure. This classification resulted in the creation of four distinct groups: Normal BMI with High Blood Pressure, Normal BMI with Low Blood Pressure, Overweight BMI with High Blood Pressure, and Overweight BMI with Low Blood Pressure [89].

Table 14 presents the distribution of fatty liver across BMI and blood pressure categories, highlighting differences, especially within the normal BMI group by blood pressure.

Individuals with an overweight BMI exhibit a high likelihood of fatty liver irrespective of blood pressure level, with rates above 90% in both high and low BP categories. This pattern suggests that within the overweight group, blood pressure does not appear to be a significant predictor of fatty liver, underscoring BMI as a more influential factor in fatty liver presence.

As expected, individuals with a normal BMI show substantially lower rates of fatty liver, especially those with low blood pressure, where only 5% have fatty liver. This pattern strengthens the idea that BMI may have a more significant impact than blood pressure on the presence of fatty liver. However, a moderate association is noted in the normal BMI group with high blood pressure, where 10% are affected by fatty liver, hinting that elevated blood pressure in otherwise normal-weight individuals could still be related to fatty liver risk, albeit to a lesser extent [89].

Based on pairwise comparisons in logistic regression (Table 15), there appear to be potential differences in fatty liver risk within the normal BMI group by blood pressure level. However, due to the small sample size, statistical significance could not be determined, as reflected by the wide confidence intervals and high *p*-values. This suggests that while there may be some association, the current data is insufficient to confirm a meaningful effect. Further investigation with a larger, balanced sample would be necessary to reliably assess whether blood pressure significantly impacts fatty liver risk within the normal BMI category [89,90].

## 9. Conclusions

This study highlights a significant association between low SHBG levels and the presence of fatty liver disease (MAFLD), demonstrating that SHBG serves as an important indicator of fatty liver in a metabolically compromised population. Individuals with fatty liver exhibited significantly lower SHBG levels compared to healthy participants, confirming SHBG’s potential value in assessing metabolic health. However, it is important to emphasize that SHBG acts as an indicator rather than a predictor, as the observed associations are based on individuals already diagnosed with fatty liver. While SHBG shows potential as a biomarker for MAFLD, its feasibility for widespread clinical use, including cost-effectiveness and accessibility, remains to be evaluated. Future research should address these factors to better understand how SHBG could be incorporated into clinical practice, particularly in resource-limited settings. Body mass index (BMI) and age also strongly influenced SHBG levels. In younger individuals, higher BMI was associated with lower SHBG levels, while in older participants, SHBG levels remained stable regardless of BMI. Elevated estradiol and HDL cholesterol levels were associated with higher SHBG levels, whereas lower SHBG levels were observed in individuals with higher triglyceride levels and fatty liver. These findings indicate a complex interplay between hormonal status, lipid profiles, and metabolic health, underscoring the role of SHBG in these relationships. While our findings are relevant to the male population, future research should explore these associations in women to provide a comprehensive understanding of gender differences in MAFLD. Although the prevalence of MAFLD increases with age, our focus was on a population up to 55 years old to emphasize the importance of weight management and prevention in the younger, working-age group. The hormonal status of older men may contribute to fatty liver development, which is why we focused on younger participants who are at risk of MAFLD and often fail to take proper care of their health. The loss of significance for certain predictors, such as glucose and HDL, when adjusting for others in the model may be attributed to multicollinearity or confounding effects. This interaction highlights the complex relationships between metabolic factors and suggests the need for further investigation into their independent and combined effects in future research.

Our research contributes to identifying SHBG as a potential biomarker for fatty liver disease and metabolic health. Specifically, our study provides several key insights:

1. **SHBG as a Diagnostic Marker**: We demonstrated a statistically significant association between low SHBG levels and the presence of fatty liver, suggesting that SHBG could serve as a useful non-invasive marker for the early detection or risk assessment of fatty liver disease.
2. **Interaction Between BMI, Age, and SHBG**: Our findings highlight a novel interaction between BMI and age, revealing that SHBG levels decrease significantly with higher BMI in younger individuals while remaining stable in older individuals. This provides new insight into how metabolic health and hormonal regulation may vary across different age groups.
3. **Link Between SHBG and Metabolic Health**: The study emphasizes SHBG’s broader role in metabolic disorders, showing its relationship not only to liver health but also to lipid profiles (HDL and triglycerides) and hormonal factors (estradiol, testosterone). This reinforces the potential utility of SHBG in assessing overall metabolic health.
4. **Impact of Blood Pressure on Fatty Liver**: Our analysis found that while both systolic and diastolic blood pressure levels were initially associated with an increased risk of MAFLD, their significance diminished when adjusted for BMI. This indicates that while elevated blood pressure may have some association with fatty liver disease, BMI remains the primary predictor of fatty liver risk. Among individuals with normal BMI, however, elevated blood pressure was linked to a slightly higher incidence of fatty liver, suggesting that blood pressure might still play a secondary role in fatty liver risk within this subgroup.
5. **Risk Factors for Fatty Liver**: In addition to SHBG, our research identifies BMI and age as primary risk factors for fatty liver disease, with blood pressure potentially influencing fatty liver prevalence within specific BMI categories. These findings contribute to a better understanding of the multifactorial nature of MAFLD and the role of metabolic markers in predicting disease progression.

The insights from this study on SHBG, BMI, and blood pressure emphasize the importance of considering a range of metabolic factors when assessing fatty liver risk. Although BMI is the dominant predictor, blood pressure’s secondary role—especially among individuals with a normal BMI—suggests potential benefits of blood pressure management in reducing fatty liver risk in select populations. This highlights the value of incorporating comprehensive metabolic assessments in clinical practice to improve early detection and risk stratification for MAFLD. Further research into these relationships could deepen understanding of the underlying mechanisms and potential interventions. This study lays the groundwork for future research to explore the effects of interventions like weight loss or hormone replacement therapy on SHBG levels and to expand these findings to other populations, such as women, where hormonal changes could play a significant role in MAFLD development.

Our study advances knowledge in the field by linking SHBG levels, BMI, and blood pressure to fatty liver and metabolic health, with implications for clinical practice and further research on metabolic disorders. While SHBG has shown promise as a biomarker for MAFLD, its integration into standard diagnostic or screening protocols will require further validation. Future clinical pathways could incorporate SHBG testing as part of routine blood work, alongside other metabolic markers, to aid in the early detection and management of MAFLD. This would require both standardization of SHBG measurement and an assessment of its cost-effectiveness, particularly in resource-limited settings.

### Limitations and Future Directions

While this study provides important insights, several limitations must be acknowledged:

- The use of ultrasonography instead of biopsy, FibroScan, or elastography for MAFLD diagnosis may limit the precision of liver fat assessment.
- SHBG’s diagnostic thresholds for MAFLD require further validation in broader, more diverse populations.
- Longitudinal studies are necessary to determine whether low SHBG levels precede fatty liver development and its associated complications.
- The study population included only male participants. Although this was intentional to avoid confounding by hormonal replacement therapies and to address a gap in male-specific data, it limits the generalizability of findings to females or mixed-gender populations. Future research should validate these findings in diverse cohorts, including both genders.

## Figures and Tables

**Table 1:** Overall descriptive statistics for the study population (N = 172).

Variable Label	Mean	Std Dev	Median	Minimum	Maximum
ALT (U/L)	36.14	23.5	31	12	210
AST (U/L)	29.11	15.83	25.5	15	173
GGT (U/L)	38.12	42.05	26	6	362
Prostate Volume (mL)	21.85	7.67	20.1	8.1	53
Body Mass Index	27.49	4.62	26.45	18.9	47.32
Waist Circumference (cm)	93.7	16.44	94	58	160
Weight (kg)	90.86	16.59	87	60	160
Height (cm)	181.6	7.25	181	166	205
Glucose (mmol/L)	5.67	0.76	5.6	4.5	11.4
Insulin (pmol/L)	85.26	53.92	73.35	14.1	413
Triglycerides (mmol/L)	1.79	1.3	1.4	0.4	9.6
Cholesterol (mmol/L)	5.47	1.03	5.3	3	8.9
HDL (mmol/L)	1.27	0.33	1.3	0.6	2.4
LDL (mmol/L)	3.53	0.93	3.35	1.5	6.4
Creatinine (µmol/L)	93.03	11.25	92	56	124
HOMA2	1.62	0.98	1.4	0.38	7.09
C-Reactive Protein (mg/L)	2.18	2.77	1.15	0.1	19
SHBG (nmol/L)	33.23	14.19	31.15	8.7	95.7
Testosterone (nmol/L)	16.78	6.06	16.07	5.25	38.55
Free Testosterone (pmol/L)	348.57	100.31	331.2	117.2	729.5
Estradiol (pmol/L)	124.19	33.58	119	52	226
Age	43.43	7.12	43	29	61

**Table 2:** Descriptive statistics for individuals with and without Metabolic-Associated Fatty Liver Disease (MAFLD).

MAFLD	N Obs	Variable Label	Mean	Std Dev	Median	Minimum	Maximum
healthy	74	ALT (U/L)	28.01	11.26	25	12	56
		AST (U/L)	26.41	9.76	24	16	73
		GGT (U/L)	22.41	16.98	17.5	6	115
		Prostate Volume (mL)	20.49	6.59	19.95	8.1	48
		Body Mass Index	23.53	1.36	23.65	18.9	26.9
		Waist Circumference (cm)	78.8	8.5	79	58	94
		Weight (kg)	77.82	7.74	78	60	102
		Height (cm)	181.69	6.86	182	168	205
		Glucose (mmol/L)	5.53	0.65	5.5	4.5	9.7
		Insulin (pmol/L)	62.01	30.63	52.8	14.1	167
		Triglycerides (mmol/L)	1.34	0.76	1.15	0.4	5.2
		Cholesterol (mmol/L)	5.25	1.05	5.05	3	8.9
		HDL (mmol/L)	1.36	0.34	1.3	0.8	2.2
		LDL (mmol/L)	3.31	0.87	3.25	1.7	6.4
		Creatinine (µmol/L)	91.46	10.27	90	56	115
		HOMA2	1.18	0.57	1.03	0.38	3.14
		C-Reactive Protein (mg/L)	1.77	2.68	0.85	0.2	17.1
		SHBG (nmol/L)	38.65	15.8	37.1	11.6	95.7
		Testosterone (nmol/L)	18.96	6.42	18.31	7.48	38.55
		Free Testosterone (pmol/L)	368.03	101.17	344.55	166.7	610.7
		Estradiol (pmol/L)	120.74	30.9	116	62	193
		Age	41.53	7.12	41.5	29	56
masna jetra	98	ALT (U/L)	42.28	28.1	37	13	210
		AST (U/L)	31.15	18.98	27	15	173
		GGT (U/L)	49.98	50.69	34	7	362
		Prostate Volume (mL)	22.87	8.28	21	10	53
		Body Mass Index	30.48	3.91	30.2	21.8	47.32
		Waist Circumference (cm)	104.96	11.17	104	85	160
		Weight (kg)	100.7	14.57	100	69	160
		Height (cm)	181.54	7.57	180	166	200
		Glucose (mmol/L)	5.77	0.83	5.65	4.6	11.4
		Insulin (pmol/L)	102.82	60.79	90.15	21.5	413
		Triglycerides (mmol/L)	2.12	1.51	1.7	0.4	9.6
		Cholesterol (mmol/L)	5.64	0.99	5.5	3	8.1
		HDL (mmol/L)	1.21	0.3	1.2	0.6	2.4
		LDL (mmol/L)	3.69	0.95	3.5	1.5	6.3
		Creatinine (µmol/L)	94.21	11.85	93	68	124
		HOMA2	1.95	1.09	1.75	0.4	7.09
		C-Reactive Protein (mg/L)	2.48	2.81	1.6	0.1	19
		SHBG (nmol/L)	29.14	11.3	28.4	8.7	68.5
		Testosterone (nmol/L)	15.13	5.22	14.42	5.25	30.93
		Free Testosterone (pmol/L)	333.87	97.61	322.85	117.2	729.5
		Estradiol (pmol/L)	126.79	35.4	121.5	52	226
		Age	44.87	6.81	45	29	61

**Table 3:** Moderate and High Correlations Between Variables (significant at α = 0.05).

Variable 1	Variable 2	Correlation Coefficient
Insulin (pmol/L)	HOMA2	0.99754
Cholesterol (mmol/L)	LDL (mmol/L)	0.93627
Weight (kg)	Waist Circumference (cm)	0.92106
Weight (kg)	Body Mass Index	0.89033
Body Mass Index	Waist Circumference (cm)	0.88206
AST (U/L)	ALT (U/L)	0.81024
Testosterone (nmol/L)	Free Testosterone (pmol/L)	0.79902
Testosterone (nmol/L)	SHBG (nmol/L)	0.74944
ALT (U/L)	GGT (U/L)	0.53299
Body Mass Index	A_HOMA2	0.47077
Body Mass Index	Insulin (pmol/L)	0.45999
Weight (kg)	HOMA2	0.43944
Waist Circumference (cm)	HOMA2	0.42235
Weight (kg)	Insulin (pmol/L)	0.42215
Height (cm)	Weight (kg)	0.41226
Waist Circumference (cm)	Insulin (pmol/L)	0.4057

**Table 4:** Univariate Linear Regression Results for SHBG with Various Predictors.

Predictor	Estimate	*p*-Value
BMI	−0.66697	**0.0042**
Height (cm)	−0.09462	0.5287
Weight (kg)	−0.19622	**0.0025**
Waist Circumference (cm)	−0.2283	**0.0005**
Age	0.176139	0.2489
Estradiol (pmol/L)	0.104326	**0.0011**
Free Testosterone (pmol/L)	0.030509	**0.0045**
Testosterone (nmol/L)	1.756273	**0**
LDL (mmol/L)	0.303421	0.7953
HDL (mmol/L)	10.99701	**0.0009**
ALT (U/L)	−0.12801	**0.0052**
Cholesterol (mmol/L)	0.409042	0.6993
Creatinine_mmol_L	−0.03294	0.7338
Prostate Volume (ml)	−0.07604	0.5925
log_HOMA2	−6.39361	**0.001**
log_Glucose	−5.00689	0.6014
log_Insulin	−6.34992	**0.0008**
log_Triglycerides	−7.85973	**0**
log_AST	−4.84818	0.1238
log_CRP	−0.70236	0.5154
log_GGT	−4.20755	**0.006**

**Table 5:** SHBG Levels in ‘Healthy’ vs. ‘Fatty Liver’ Individuals (*t*-test results)-Pooled and Satterthwaite Method.

Indicator	Method	Mean	95% CL Mean		Std Dev	95% CL Std Dev	
healthy		38.6541	34.9924	42.3158	15.8049	13.605	18.8601
fatty liver		29.1418	26.876	31.4077	11.3017	9.9107	13.1507
Diff (1-2)	Pooled	9.5122	5.4319	13.5926	13.4218	12.1342	15.0177
Diff (1-2)	Satterthwaite	9.5122	5.2316	13.7929			

**Table 6:** SHBG Levels in ‘Healthy’ vs. ‘Fatty Liver’ Individuals (*t*-test results). Descriptive statistics for each group and for differences between groups.

Method	Variances	DF	*t* Value	Pr > |t|
Pooled	Equal	170	4.6	<0.0001
Satterthwaite	Unequal	126.11	4.4	**<0.0001**

**Table 7:** Folded F Test for Equality of Variances: SHBG by MAFLD.

Equality of Variances				
Method	Num DF	Den DF	F Value	Pr > F
Folded F	73	97	1.96	**0.0021**

**Table 8:** GLM Parameter Estimates for Full Model.

Pr > |t|	*t* Value	Standard	Estimate	Parameter
		Error		
0.0667	1.85	44.81564	82.74983	Intercept
**0.0009**	−3.39	3.230603	−10.94868212	indicator fatty liver
.	.	.	0	indicator healthy
0.0408	−2.06	1.45294	−2.99730235	BMI
0.0556	−1.93	0.882244	−1.70189968	Age
**0.0191**	2.37	0.032246	0.076347	BMI * Age
0.9611	−0.05	0.128335	−0.00627478	Prostate_Volume_ml
**0.0078**	2.7	1.138471	3.070619	Cholesterol_mmol_L
0.9102	−0.11	2.187118	−0.24710276	log_Homa2
0.6979	0.39	4.217865	1.640151	HDL_mmol_L
0.9417	0.07	0.09024	0.006605	Creatinine_mmol_L
0.606	−0.52	3.302265	−1.70671827	log_AST
0.8301	0.21	1.131073	0.243094	log_CRP
0.7472	0.32	1.942321	0.62717	log_GGT
0.612	−0.51	9.285214	−4.71960208	log_Glucose
**0.0168**	−2.42	2.604745	−6.29505522	log_Triglycerides
**0.0178**	2.4	0.03376	0.080872	Estradiol_pmol_L
0.1263	1.54	0.011997	0.018441	Free_Testosterone_pm

* Interaction between BMI and Age.

**Table 9:** General Linear Model—Stepwise Selection Summary for the Chosen Model.

Stepwise Selection Summary							
Step	Effect Entered	Effect Removed	Number Effects In	Number Parms In	SBC	F Value	Pr > F
0	Intercept		1	1	912.148	0	1
1	indicator		2	2	897.386	20.86	<0.0001
2	Estradiol_pmol_L		3	3	886.495	16.51	<0.0001
3	HDL_mmol_L		4	4	885.9338	5.66	0.0184
4	BMI * Age		5	5	885.8117	5.19	0.024
5	Free_Testosterone_pm		6	6	885.3873 *	5.46	0.0207

* Optimal Value of Criterion.

**Table 10:** General Linear Model—Parameter Estimates for the Chosen Model.

Parameter Estimates				
Parameter	DF	Estimate	Standard Error	*t* Value
Intercept	1	−4.685729	7.994332	−0.59
indicator fatty liver	1	−12.801731	2.564916	−4.99
indicator healthy	0	0	.	.
BMI * Age	1	0.013176	0.004484	2.94
HDL_mmol_L	1	8.85366	3.067382	2.89
Estradiol_pmol_L	1	0.073817	0.032099	2.3
Free_Testosterone_pm	1	0.025815	0.011049	2.34

* Interaction between BMI and Age.

**Table 11:** Logistic Regression Model: Summary of Stepwise Selection.

Summary of Stepwise Selection								
Step	Effect		DF	Number In	Score Chi-Square	Wald Chi-Square	Pr > ChiSq	Variable Label
	Entered	Removed						
1	BMI		1	1	95.8713		<0.0001	Body Mass Index
2	SHBG_nmol_L		1	2	5.3406		0.0208	SHBG (nmol/L)
3	Age		1	3	4.7245		0.0297	Age
4	log_Glucose		1	4	7.8293		0.0051	Log of Glucose (mmol/L)

**Table 12:** Odds Ratios and Wald Confidence Intervals.

Odds Ratio Estimates and Wald Confidence Intervals			
Odds Ratio	Estimate	95% Confidence Limits	
SHBG_nmol_L	0.875	0.786	0.974
BMI	6.048	2.663	13.738
Age	1.186	1.043	1.349
log_Glucose	<0.001	<0.001	0.065

**Table 13:** Logistic Regression Results (Uni and Multiple).

	Univariate		Multiple (Systolic, BMI)		Multiple (Diastolic, BMI)		Multiple (Systolic, Diastolic, BMI)	
Predictor	Wald Chi-Square	Pr > ChiSq	Wald Chi-Square	Pr > ChiSq	Wald Chi-Square	Pr > ChiSq	Wald Chi-Square	Pr > ChiSq
BMI	30.1470	<0.0001	29.0445	<0.0001	28.6570	<0.0001	28.5889	<0.0001
DIASTOLIC	18.6441	<0.0001			0.4858	0.4858	0.1773	0.6737
SYSTOLIC	14.0008	0.0002	0.3291	0.5662			0.0137	0.9069

**Table 14:** Table of combined groups by MAFLD.

Combined_Group	Indicator (MAFLD)		
Frequency Row Pct	Healthy	Fatty Liver	Total
Normal BMI-High BP	9 90.00	1 **10.00**	10
Normal BMI-Low BP	57 95.00	3 **5.00**	60
Overweight BMI-High BP	3 7.32	38 92.68	41
Overweight BMI-Low BP	5 8.20	56 91.80	61
Total	74	98	172

**Table 15:** Specific Pairwise Comparisons (Logistic Regression).

Label	Estimate	Pr > |z|	Exponentiated	Exponentiated Lower	Exponentiated Upper
Normal BMI-High BP vs. Normal BMI-Low BP	0.7472	0.5366	2.1111	0.1974	22.5799
Overweight BMI-High BP vs. Overweight BMI-Low BP	0.1231	0.8714	1.1310	0.2550	5.0154

## Data Availability

The original contributions presented in this study are included in the article. Further inquiries can be directed to the corresponding author(s).
